# Cd1d regulates B cell development but not B cell accumulation and IL10 production in mice with pathologic CD5^+^ B cell expansion

**DOI:** 10.1186/s12865-015-0130-z

**Published:** 2015-11-04

**Authors:** Victoria L. Palmer, Vincent K. Nganga, Mary E. Rothermund, Greg A. Perry, Patrick C. Swanson

**Affiliations:** Department of Medical Microbiology and Immunology, Creighton University, 2500 California Plaza, Omaha, NE 68178 USA

**Keywords:** CD1d, CLL, B10 B cell, Natural killer T cell, CD5, TCL1

## Abstract

**Background:**

CD1d is a widely expressed lipid antigen presenting molecule required for CD1d-restricted invariant natural killer T (iNKT) cell development. Elevated CD1d expression is detected in CD5^+^ IL10-producing B cells, called B10 B cells, and is correlated with poorer prognosis in chronic lymphocytic leukemia (CLL), a CD5^+^ B cell malignancy with B10-like functional properties. Whether CD1d expression regulates CD5^+^ B cell accumulation, IL10 competence, and antibody production in naïve mice with pathologic CD5^+^ B cell expansion remains untested.

**Results:**

Using three different transgenic mouse models of benign or leukemic CD5^+^ B cell expansion, we found that CD1d was differentially expressed on CD5^+^ B cells between the three models, but loss of CD1d expression had no effect on CD5^+^ B cell abundance or inducible IL10 expression in any of the models. Interestingly, in the CLL-prone Eμ-TCL1 model, loss of CD1d expression suppressed spontaneous IgG (but not IgM) production, whereas in the dnRAG1xEμ-TCL1 (DTG) model of accelerated CLL, loss of CD1d expression was associated with elevated numbers of splenic CD4^+^ and CD8^+^ T cells and an inverted CD4^+^:CD8^+^ T cell ratio. Unexpectedly, before leukemia onset, all three transgenic CD1d-deficient mouse strains had fewer splenic transitional B cells than their CD1d*-*proficient counterparts.

**Conclusions:**

The results show that CD1d expression and iNKT cells are dispensable for the development, accumulation, or IL10 competence of CD5^+^ B cells in mice prone to benign or leukemic CLL-like B cell expansion, but reveal a novel role for iNKT cells in supporting B cell progression through the transitional stage of development in these animals. These results suggest CD1d-directed therapies to target CLL could be evaded by downregulating CD1d expression with little effect on continued leukemic CD5^+^ B cell survival. The data also imply that iNKT cells help restrain pro-leukemic CD8^+^ T cell expansion in CLL, potentially explaining a reported correlation in human CLL between disease progression, the loss of NKT cells, and a paradoxical increase in CD8^+^ T cells.

**Electronic supplementary material:**

The online version of this article (doi:10.1186/s12865-015-0130-z) contains supplementary material, which is available to authorized users.

## Background

CD1d is a non-polymorphic MHC-like molecule that functions to present lipid-based antigens to a subset of T cells expressing natural killer cell lineage markers and a CD1d-restricted T cell receptor. Most of these CD1d-restricted T cells express a T cell receptor comprised of a semi-invariant TCRα chain paired with a member of a restricted set of TCRβ chains, and are therefore termed invariant natural killer T (iNKT) cells [1]. In humans, CD1d is one member of a larger family of CD1 molecules that also includes CD1a, CD1b, CD1c, and CD1e; mice only express CD1d and completely lack orthologs for the other four CD1 isoforms [1]. CD1d is expressed on most normal B cells [2], but more highly so on certain B cell subsets, including marginal zone B cells [3] and a population of regulatory B cells, called B10 B cells, that produce IL10 after mitogenic stimulation [4]. CD1d is also broadly expressed in various B cell chronic lymphoproliferative disorders (B-CLPDs): CD1d expression on leukemic B cells in chronic lymphocytic leukemia (CLL) is generally lower than on other B-CLPDs (such as mantle cell lymphoma) [5], but elevated CD1d expression in CLL has been associated with the presence of unmutated immunoglobulin variable region genes [6] and poor prognosis [7], suggesting its potential utility as a biomarker for this disease. Interestingly, a recent report established that murine and human CLL cells, like B10 cells, are also IL10-competent [8].

iNKT cells in mice and humans respond to similar antigens in vivo and fulfill similar immunological roles in both species, although subtle differences in the number, distribution, and effector function of iNKT cell populations have been described (for review, see [9]). iNKT cells play an important role in promoting protective antibody responses (for review, see [10]). On the other hand, iNKT cells also function to suppress B cell autoreactivity [11, 12]. Paradoxically however, iNKT cells have been shown to enhance spontaneous autoantibody secretion by B cells from patients with systemic lupus erythematosus (SLE) [13], and murine models of this disease [14]. iNKT cells fail to develop in *Cd1d*^−/−^ mice [15], but defects in B cell development have not been reported in these animals.

Previously, we described transgenic mice expressing catalytically inactive RAG1 (dnRAG1 mice), which develop a benign early-onset CD5^+^ B cell lymphocytosis with hypogammaglobulinemia, and exhibit a restricted B cell receptor repertoire with biased Jκ1 usage consistent with a defect in B cell receptor editing [16]. The indolent accumulation of CD5^+^ B cells in dnRAG1 mice is reminiscent of monoclonal B cell lymphocytosis (MBL), a condition that generally precedes the onset of CLL [17]. Consistent with this hypothesis, dnRAG1 transgene expression in the well-established Eμ-TCL1 model of murine CLL was found to significantly accelerate disease progression [18]. Given recent evidence that murine and human CLL possess B10-like phenotypic and functional properties [8], we hypothesized that CD5^+^ B cells in dnRAG1 mice and dnRAG1/Eμ-TCL1 double-transgenic (DTG) mice functionally resemble B10 B cells. We further postulated that CD1d expression and iNKT cells are required for proper regulation of CD5^+^ B cell accumulation, IL10 production, and spontaneous antibody levels in these animals.

To test these hypotheses, we analyzed CD1d expression levels in wild-type, dnRAG1, Eμ-TCL1, and DTG mice, and compared the absolute number of CD5^+^ B cells, the frequency of CD5^+^IL10^+^ B cells after mitogenic stimulation in vitro, and the concentration of serum IgM and IgG in naïve wild-type, dnRAG1, Eμ-TCL1, and DTG mice on a CD1d-proficient or a CD1d-deficient strain background. Our findings reveal that CD5^+^ B cells in dnRAG1 and DTG mice express higher levels of CD1d than both CD5^+^ B cells from Eμ-TCL1 mice and CD5^−^ B cells from wild-type mice. Nevertheless, CD5^+^ B cell accumulation and IL10-competence in dnRAG1, Eμ-TCL1, and DTG mice were not significantly altered by loss of CD1d expression and iNKT cells. Interestingly, at 12 weeks of age before leukemia onset in CLL-prone mouse strains, dnRAG1, Eμ-TCL1, and DTG mice all showed significantly fewer transitional B cells in the CD1d-deficient strain background compared to their CD1d-proficient counterparts. In addition, loss of CD1d expression in Eμ-TCL1 mice led to a significant reduction in serum IgG. Furthermore, older CD1d-deficient DTG mice showed significant increases in CD4^+^ and CD8^+^ T cells and an inverted CD4^+^:CD8^+^ T cell ratio. Taken together, these data suggest that CD1d expression and iNKT cells are dispensable for B10-like CD5^+^ B cell accumulation and IL10-competence in mice prone to MBL-like and CLL-like disorders, but iNKT cells play a previously unrecognized role in regulating B cell progression through the transitional stages of B cell development. Furthermore, iNKT cells may promote spontaneous antibody production in Eμ-TCL1 mice and regulate T cell expansion in DTG mice.

## Methods

### Mice

Transgenic dnRAG1, Eμ-TCL1, and DTG (dnRAG1/Eμ-TCL1 double-transgenic) mice, all on C57Bl/6 backgrounds, have been described previously [16, 18, 19]. Mice harboring a conditional (floxed) *Cd1d1* allele on a C57Bl/6 background [20] (called *Cd1d*^fl^ henceforth) were obtained from the Jackson Laboratory (*Cd1d1*/*Cd1d2*^*tm1.1Aben*^/J; note that *Cd1d2* is a pseudogene in the C57Bl/6 background [21]). Our original intent was to use mb1-Cre transgenic mice [22] to generate conditional *Cd1d* knock-out mice in wild-type, dnRAG1, Eμ-TCL1, and DTG strain backgrounds to evaluate how selective loss of CD1d expression in B cells affects CD5^+^ B cell accumulation and functionality in the different strain backgrounds. However, due to unexpected Cre-mediated germline deletion of the *Cd1d*^fl^ allele, this approach was not feasible. As an alternative, we bred out the mb1-Cre transgene, and intercrossed mice harboring the germline-deleted *Cd1d* allele to generate *Cd1d*^del/del^ mice. The dnRAG1 and Eμ-TCL1 transgenes were then bred individually onto the *Cd1d*^del/del^ background, and the two strains were intercrossed to obtain cohorts of *Cd1d*^del/del^ wild-type (WT), *Cd1d*^del/del^ dnRAG1, *Cd1d*^del/del^ Eμ-TCL1, and *Cd1d*^del/del^ DTG mice. These animals were euthanized at 12 or 36 weeks of age and analyzed in parallel to age-matched *Cd1d*^+/+^ mice with the same genotype in the experiments described below. Thus, rather than being a conditional model of *Cd1d* gene disruption, *Cd1d* expression is disrupted in all cell lineages in *Cd1d*^del/del^ mice. All mice were housed in individually ventilated microisolator cages in an AAALAC certified animal facility in accordance with university and federal guidelines, and mouse protocols were approved by the Creighton University Institutional Animal Care and Use Committee. See Additional file 1 for further details.

### Flow cytometry

Single-cell suspensions prepared from spleen and bone marrow were depleted of red blood cells by hypotonic lysis, stained with fluorochrome-conjugated antibodies or CD1d tetramers, and subjected to flow cytometric analysis as described in Additional file 1.

### Analysis of IL10 production

Intracellular IL10 protein induction following in vitro stimulation with lipopolysaccharide (LPS), phorbol-12-myristate-13-acetate (PMA), ionomycin, and monensin (LPS + PIM) was detected by flow cytometry as described previously [4, 23]. Briefly, 10^6^ splenocytes, depleted of red blood cells, were incubated in complete medium (RPMI 1640 medium containing 10 % FCS, 200 μg/ml penicillin, 200 U/ml streptomycin, 4 mM L-glutamine, and 50 μM 2-mercaptoethanol; 100 μL final volume) with LPS (10 μg/ml, *Escherichia coli* serotype 0111:B4; Sigma-Aldrich), PMA (50 ng/ml; Sigma- Aldrich), ionomycin (1 μg/ml; Sigma-Aldrich), and monensin (2 μM; eBioscience) for 4 h, in 96 well flat-bottom plates. As a control, some samples were treated with only monensin. For IL-10 detection, cells were treated with Fc-block reagent (anti-CD16/CD32, clone 93; eBioscience) before cell surface staining. Stained cells were fixed and permeabilized using a Cytofix/Cytoperm kit (BD Pharmingen), according to the manufacturer’s instructions, and stained with APC-conjugated mouse anti-IL-10 mAb (JES5-16E3; eBioscience) or isotype matched control (eB149/10H5; eBioscience).

### Immunoglobulin levels

Serum Igs were measured by ELISA with IMMUNO-TEK mouse IgM and IgG kits (ZeptoMetrix, Buffalo, NY) according to manufacturer’s instructions. Optical density was measured with VersaMax microplate reader (Molecular Devices, Sunnyvale, CA).

### Statistics

Collected data were subjected to analysis of variance and post hoc testing using the PASW Statistics 22.0 software. A *p* value < 0.05 was considered significant. Data is presented as mean ± the standard error of the means.

## Results

### CD5^+^ B cells in dnRAG1 and DTG mice express elevated levels of CD1d

**Fig. 1:**
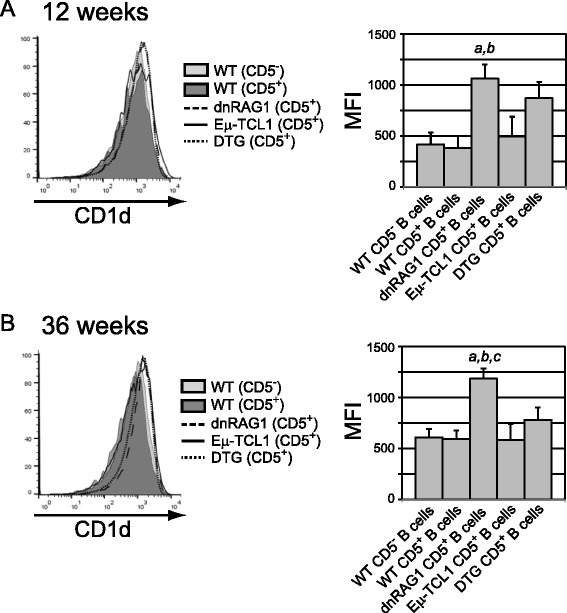
CD5^+^ B cells in dnRAG1 and DTG mice express high levels of CD1d. **a**-**b** Cohorts of 12 week-old (**a**) and 36 week-old (**b**) mice were analyzed for CD1d expression on CD19^+^ and CD5^−^ or CD5^+^ B cells from WT, dnRAG1, Eμ-TCL1 and DTG mice. (*Left Panel*) Overlay of CD1d expression on CD19^+^CD5^−^ (WT) or CD19^+^CD5^+^ (WT, dnRAG1, Eμ-TCL1 and DTG) B cells from representative animals. (*Right panel*) Mean fluorescence intensity of CD1d staining in the gated populations in the left panel (*n* = 4–6 animals/genotype). Statistically significant differences (*p* < 0.05) were only detected in comparisons between dnRAG1 mice and the other mouse genotypes (*a*, vs WT mice; *b*, vs Eμ-TCL1 mice; *c*, vs DTG mice)

Although the CD5^+^ B cells accumulating in dnRAG1 mice and DTG mice phenotypically resemble B10 B cells insofar as CD5 expression is concerned, unlike the B10 B cells described in early studies [4], they do not express CD21 [18]. We nevertheless considered the possibility that they functionally resemble B10 B cells for several reasons. First, previous gene expression profiling analysis of CD5^+^ B cells from dnRAG1, Eμ-TCL1, and DTG mice showed that these cells all express IL10 at higher levels than CD5^−^ B cells from WT mice [18]. Second, a recent study by DiLillo et al*.* showed that human CLL cells and CLL-like CD5^+^ B cells from Eμ-TCL1 mice, which we have shown to be CD21^−^ [18], can be stimulated to express IL10 by LPS + PIM treatment in vitro [8]. Third, analysis of the gene expression data from our previous study [18] provided evidence that *Cd1d* expression is higher in CD5^+^ B cells from dnRAG1 mice and DTG mice than either CD5^−^ B cells from WT mice or CD5^+^ B cells from Eμ-TCL1 mice at 12 and 36 weeks of age. These time points were chosen for the following reasons: (a) at 12 weeks of age, CD5^+^ B cells represent a large fraction of splenic B cells in dnRAG1 and DTG mice, but only a small fraction of splenic B cells in WT and Eμ-TCL1 mice; and (b) the 36 week time point represents the approximate median life span of DTG mice where CLL onset is evident [18]. Flow cytometric analysis confirmed that at both time points, surface CD1d protein levels on CD5^+^ B cells from dnRAG1 mice and DTG mice were elevated compared to CD5^−^ and CD5^+^ B cells from WT mice or CD5^+^ B cells from Eμ-TCL1 mice (Fig. 1).

### CD5^+^ B cells from wild-type and transgenic mice are IL10 competent

**Fig. 2:**
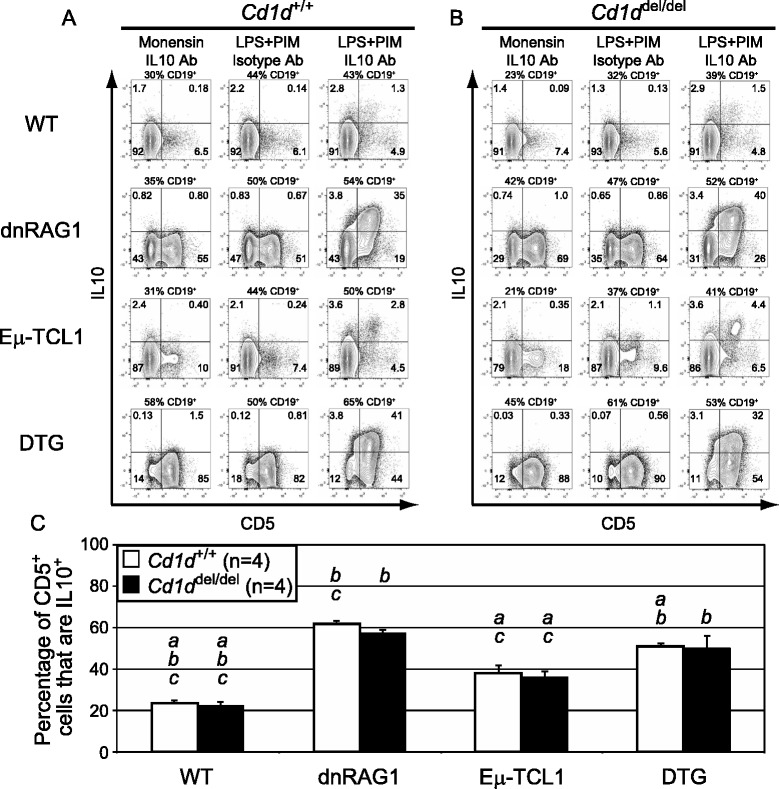
CD5^+^ B cells in 12 week-old dnRAG1 and DTG mice support inducible IL10 expression in vitro, but IL10 competence is CD1d-independent. **a**-**b** Splenocytes from 12 week-old wild-type, dnRAG1, Eμ-TCL1, and DTG mice on a *Cd1d* ^*+/+*^ (**a**) or *Cd1d* ^*del/del*^ (**b**) background were incubated with monensin alone or LPS + PIM for 4 h. After permeabilization, the cells were stained with IL-10-specific or isotype-control monoclonal antibody (Ab) as indicated. Gated CD19^+^ B cells were analyzed for CD5 and IL10 expression. The percentage of cells in each quadrant is shown for representative animals. **c** The mean percentage of total CD5^+^ B cells staining positive for IL10 for *n* = 4 mice/genotype is plotted in bar graph format. Error bars represent the SEM. Statistically significant (*p* < 0.05) differences between groups of WT and transgenic animals with the same *Cd1d* genotype are indicated (*a*, vs dnRAG1; *b*, vs Eμ-TCL1; *c*, vs DTG). No significant differences between *Cd1d* ^*+/+*^ and *Cd1d* ^*del/del*^ animals with the same transgenes were detected

**Fig. 3:**
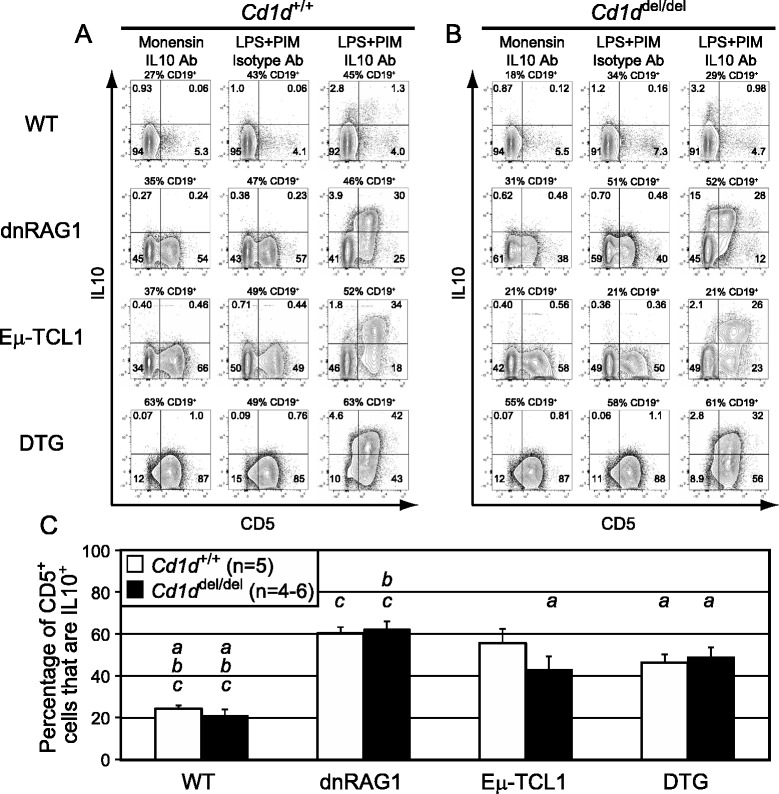
Eμ-TCL1 mice at 36 weeks of age show increased inducible IL10 expression in vitro compared to 12 weeks, but IL10 competence remains CD1d-independent. (**a-c**) Splenocytes from 36 week-old wild-type, dnRAG1, Eμ-TCL1, and DTG mice on a *Cd1d* ^*+/+*^ or *Cd1d* ^*del/del*^ background were analyzed as Fig. 2. No significant differences between *Cd1d* ^*+/+*^ and *Cd1d* ^*del/del*^ animals with the same transgenes were detected

B10 B cells were initially characterized as a splenic CD1d^hi^CD5^+^CD19^hi^ population that expresses IL10 after in vitro stimulation with LPS + PIM. Monensin inhibits secretion, allowing cytoplasmic IL10 protein to accumulate to levels detectable by flow cytometry after intracellular staining with an IL10-specific monoclonal antibody [4]. LPS enhances, but is not required for, IL10 production under these conditions [24]. Because the CD5^+^ B cells accumulating in dnRAG1 and DTG mice share the CD1d^hi^ phenotype of B10 B cells, we investigated whether these cells support activation-induced IL10 expression like B10 cells. To test this possibility, splenocytes from 12 or 36 week-old WT, dnRAG1, Eμ-TCL1, and DTG mice were stimulated with LPS + PIM for 4 h in vitro and analyzed for intracellular IL10 protein by flow cytometry as described [4, 23]. We find that among gated CD19^+^ B cells at 12 weeks of age, ~23 % of CD5^+^ B cells from WT mice stained IL10^+^ after LPS + PIM treatment. Interestingly, this value was slightly higher in Eμ-TCL1 mice (~38 %), and even more elevated in dnRAG1 and DTG mice (~62 % and 51 %, respectively) (Fig. 2a and c). CD5^−^ B cells from the same animals showed little if any inducible IL10 expression (Fig. 2a). No staining was detected using an isotype control antibody, or after monensin treatment alone. The latter result shows that, despite being upregulated at the transcript level in transgenic CD5^+^ B cells [18], IL10 protein is not expressed at sufficient levels to be detectable by flow cytometry under these conditions. Similar results were obtained at 36 weeks, although the percentage of CD19^+^CD5^+^ B cells in Eμ-TCL1 mice that stained IL10^+^ more closely resembled dnRAG1 mice at this time point than at 12 weeks of age (Fig. 3a and c). Taken together, these results establish that CD5^+^ B cells in dnRAG1 and DTG mice share the feature of IL10 competence that B10 B cells and CD5^+^ B cells in older Eμ-TCL1 mice possess.

### CD1d expression and iNKT cells are dispensable for CD5^+^ B cell accumulation and IL10 competence in dnRAG1, Eμ-TCL1, and DTG mice

The observation that CD5^+^ B cells in dnRAG1 and DTG mice accumulate more rapidly and express higher levels of CD1d than CD5^+^ B cells from Eμ-TCL1 mice caused us to question the requirement for CD1d expression and the presence of iNKT cells in the expansion and function of these cells, particularly in light of evidence suggesting a positive correlation between elevated CD1d expression levels and poor prognosis in CLL [7], and reports suggesting that NKT-like cells are reduced in progressive CLL [25, 26] (although another study found normal numbers of iNKT cells in a cohort of untreated CLL patients [27]). To directly test this possibility, we acquired mice harboring a conditional *Cd1d1* allele [20], from which a germline-deleted *Cd1d* allele was obtained to generate *Cd1d*^del/del^ mice (see Material and Methods). The dnRAG1 and Eμ-TCL1 transgenes were crossed onto the *Cd1d*^del/del^ background, and then intercrossed to obtain cohorts of *Cd1d*^del/del^ WT, dnRAG1, Eμ-TCL1, and DTG mice that were analyzed in parallel to their *Cd1d*^+/+^ counterparts.

We found that at 12 weeks of age, the absolute number of splenic CD19^+^CD5^+^ B cells in *Cd1d*^*del*/*del*^ WT, dnRAG1, Eμ-TCL1, and DTG mice was not significantly different than in their *Cd1d*^+/+^ counterparts (Additional file 2: Table S1), and these cells were similarly competent to express IL10 after LPS + PIM treatment (compare Fig. 2a to b; see Fig. 2c), despite the loss of surface CD1d on B cells and the expected absence of iNKT cells (Additional file 3: Figure S1A). Similar results were obtained for 36 week-old animals; although a trend toward increased abundance of CD5^+^ B cells and lower IL10-competence was noted in *Cd1d*^*del/del*^ Eμ-TCL1 mice compared to their *Cd1d*^+/+^ counterparts (see Additional file 4: Table S2; compare Fig. 3a to b; see Fig. 3c), the differences were not statistically significant due to greater variability in the absolute numbers of CD5^+^ B cells found in *Cd1d*^*del/del*^ Eμ-TCL1 mice at this age. Thus, we conclude from these data that CD1d and iNKT cells are dispensable for the accumulation and IL10 competence of CD5^+^ B cells in WT, dnRAG1, Eμ-TCL1, and DTG mice.

### CD1d expression and iNKT cells are required for normal progression through the transitional stage of B cell development and for regulating T cell expansion in DTG mice

**Fig. 4:**
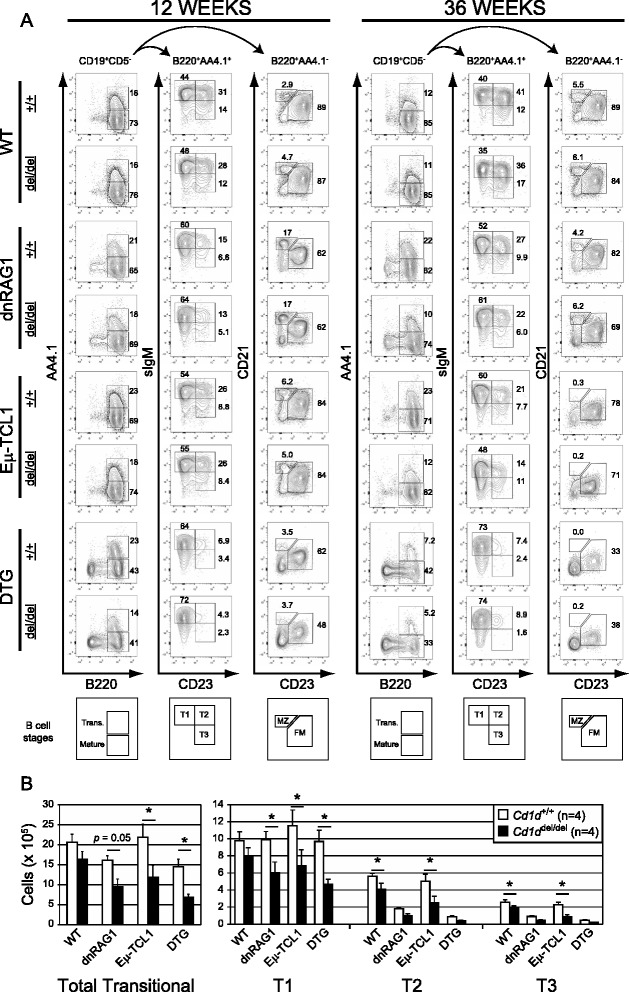
Progression through the transitional stages of B cell development is impaired in *Cd1d* ^*del/del*^ mice. **a** Gated splenic CD19^+^CD5^−^ B cells from 12 week-old or 36 week-old WT, dnRAG1, Eμ-TCL1 and DTG mice on a *Cd1d* ^+/+^ (+/+) or *Cd1d* ^del/del^ (del/del) background were initially segregated into transitional (trans.) and mature B cell subsets based on differential expression of AA4.1 and B220. Transitional (AA4.1^+^B220^hi^) and mature (AA4.1^−^B220^hi^) B cell subsets were further separated into T1-T3, marginal zone (MZ) and follicular mature (FM) populations based on differential expression of IgM, CD21, and CD23 using the gating strategy found at the bottom of the figure. The percentage of cells within the identified gates is shown for representative animals. Data collected from *n* = 4–6 animals/genotype were used to calculate the absolute number of cells in each population, and are summarized in Additional file 2: Table S1 and Additional file 4: Table S2. **b** Values for the mean absolute number of total transitional B cells (B220^hi^AA4.1^+^CD5^−^) and for each transitional B cell subset (T1-T3) are plotted in bar graph format for the 12 week time point in (**a**). Error bars represent the standard error of the mean. Statistically significant differences (*p* < 0.05) between groups of *Cd1d* ^*+/+*^ or *Cd1d* ^*del/del*^ mice with the same transgenic background are identified by an asterisk

**Fig. 5:**
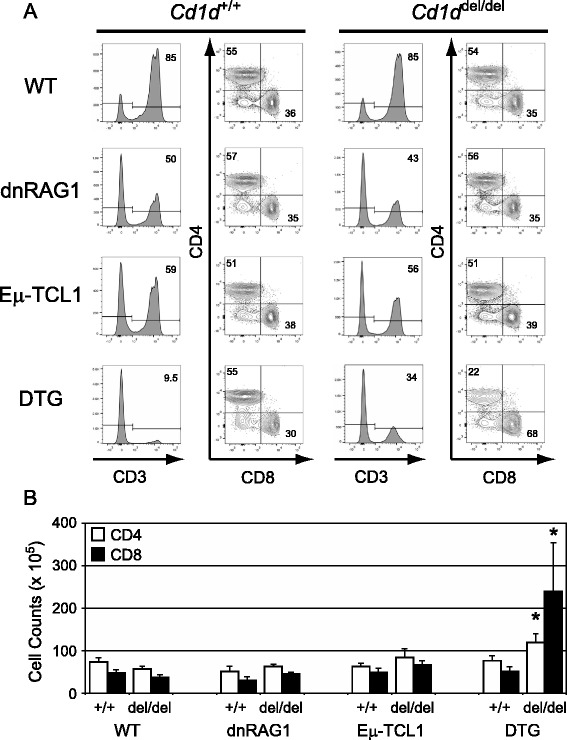
Loss of CD1d expression in 36 week-old DTG mice is associated with expansion of splenic T cells, particularly CD8^+^ T cells. **a** Gated splenic CD3^+^B220^−^ lymphocytes were analyzed for CD4 and CD8 expression. The percentage of cells within the identified gates or quadrants is shown for representative animals. Note that in DTG mice, the B220^−^ gate does not exclude all CD5^+^ B cells, which are highly abundant and express variably low levels of B220 in these animals. Data collected from *n* = 4–6 animals/genotype were used to calculate the absolute number of cells in each population, and are summarized in Additional file 4: Table S2. **b** Values for the mean absolute number of CD4^+^ and CD8^+^ T cells determined from (**a**) are plotted in bar graph format. Error bars represent the standard error of the mean. Statistically significant differences (*p* < 0.05) between groups of *Cd1d* ^*+/+*^ or *Cd1d* ^*del/del*^ mice with the same transgenic background are identified by an asterisk

Besides the loss of iNKT cells, there were two other cell populations that were significantly affected by loss of CD1d expression in these studies. The first was the B220^hi^AA4.1^+^ transitional B cell subset, which was significantly reduced (40–50 %) in 12 week-old *Cd1d*^del/del^ dnRAG1, Eμ-TCL1, and DTG mice compared to their *Cd1d*^+/+^ counterparts, due mostly to loss of transitional T1 B cells, (Fig. 4a-b; Additional file 2: Table S1). While the total number of transitional B cells was also slightly reduced in WT mice, the difference was not statistically significant at this time point. Similar trends were also noted at 36 weeks, but the differences were not statistically significant (Fig. 4a; Additional file 4: Table S2). The second was the striking increase in the abundance of CD8^+^ T cells, and to a lesser extent CD4^+^ T cells, in 36 week-old *Cd1d*^del/del^ DTG mice compared to their *Cd1d*^+/+^ counterparts that resulted in an inverted CD4:CD8 T cell ratio in *Cd1d*^del/del^ DTG mice (Fig. 5; Additional file 4: Table S2). These increases were not evident in 12 week-old DTG mice despite the abundance of CD5^+^ B cells in these animals at this time point (Additional file 2: Table S1), suggesting this outcome is correlated with disease progression in DTG mice. Thus, we conclude from these data that CD1d expression and iNKT cells are required for efficient progression through the transitional stage of B cell development, and for regulating T cell expansion in DTG mice.

### Cd1d regulates spontaneous antibody production in Eμ-TCL1 mice, but not WT, dnRAG1, or DTG mice

**Fig. 6:**
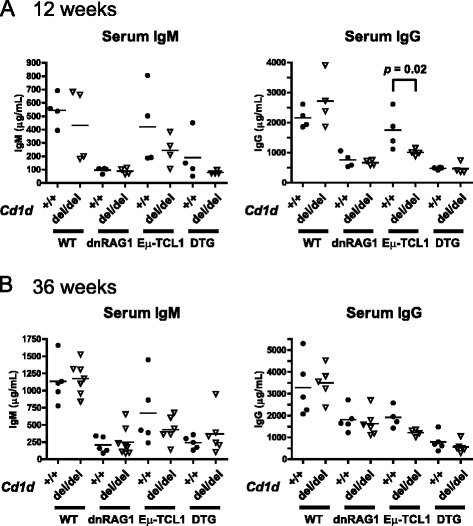
Loss of CD1d expression does not significantly affect spontaneous antibody production in dnRAG1 and DTG mice. Serum IgM and IgG was measured from 12 (**a**) and 36 (**b**) week-old WT, dnRAG1, Eμ-TCL1, and DTG mice on a *Cd1d* ^*+/+*^ or *Cd1d* ^*del/del*^ background (closed circles or open triangles, respectively). Individual values and means (identified by horizontal bar) are shown for each group; statistically significant differences between groups are indicated

In previous studies, we demonstrated that naïve dnRAG1 and DTG mice exhibit hypogammaglobulinemia, which may reflect a mechanism to restrain antibody production by B cells in which autoreactivity is enforced by a defect in receptor editing due to dnRAG1 transgene expression [18]. Given the CD1d^hi^ phenotype of the CD5^+^ B cells accumulating in these animals, and previous reports that iNKT cells play a role in suppressing B cell autoreactivity [11, 12], we hypothesized that dnRAG1 and DTG mice lacking iNKT cells would show increased spontaneous antibody production than their CD1d-proficient counterparts. However, we found that serum IgM and IgG levels between naïve *Cd1d*^del/del^ dnRAG1 and DTG mice and their *Cd1d*^+/+^ counterparts were not significantly different at either 12 or 36 weeks of age (Fig.6a-b). Notably, IgM and IgG levels in both of these backgrounds remained below those found in comparable WT mice at both time points. Therefore, we conclude that iNKT cells are not responsible for sustaining hypogammaglobulinemia observed in dnRAG1 and DTG mice. Interestingly, we note that *Cd1d*^del/del^ Eμ-TCL1 mice showed significantly reduced IgG levels compared to their *Cd1d*^+/+^ counterparts at 12 weeks of age (Fig.6a). This trend was also detected at the 36 week time point, but the difference was not statistically significant (Fig. 6b). Taken together, these data suggest a role for iNKT cells in promoting IgG production in Eμ-TCL1 mice.

## Discussion

CD1d is a widely expressed, non-polymorphic, MHC Class I-like lipid antigen-presenting molecule. CD1d expression is elevated on a normal subset of immunoregulatory CD5^+^ B cells, called B10 B cells, which have a defining feature of producing IL10 in response to stimulation by LPS + PIM in vitro. In pathological CD5^+^ B cell conditions, elevated CD1d expression has been correlated with unmutated Ig gene usage and poor prognosis in human CLL [6, 7]. Like B10 B cells, CD5^+^ cells in human CLL and the murine Eμ-TCL1 model of this disease also support inducible IL10 expression [8]. Whether CD1d expression is required to support the development and accumulation of IL10-competent, CD5^+^ B cells in a pathological context has not been formally investigated. Likewise, whether iNKT cells play a role in regulating B cell accumulation and/or spontaneous production of immunoglobulins under conditions of pathological CD5^+^ B cell expansion remain unclear.

Using three different transgenic strains of mice representing an early-onset benign CD5^+^ B cell lymphocytosis (dnRAG1 mice), an established murine model of CLL (Eμ-TCL1 mice), and a model of accelerated murine CLL progression (DTG mice), we have confirmed the earlier observation that a subset of CD5^+^ B cells in Eμ-TCL1 mice (and WT mice) support inducible IL10 production after LPS + PIM treatment in vitro [8]*,* and established here that splenic CD5^+^ B cells accumulating in dnRAG1 and DTG mice are also IL10 competent. Interestingly, the frequency of IL10^+^ cells in the CD5^+^ B cell compartment in these experiments is significantly higher for dnRAG1 and DTG mice compared to both WT and Eμ-TCL1 mice at 12 weeks of age, but at 36 weeks, the frequency of CD5^+^IL10^+^ B cells in Eμ-TCL1 mice rises so that these differences are no longer significant. Although the mechanistic basis for increasing IL10 competence among CD5^+^ B cells in Eμ-TCL1 mice remains unclear, we speculate that this observation reflects the gradual replacement of natural B10 B cells by leukemic B10-like B cells as the animal ages. Nevertheless, the data presented here clearly demonstrate that CD1d expression and the presence of iNKT cells are both dispensable for the development, accumulation, and IL10-competence of CD5^+^ B cells in all three strains of transgenic mice. Since CD1d is expressed in both mice and humans, and supports comparable immune functions in B cells in both species in vivo [1, 10], this finding may have important implications for the potential success of therapeutic strategies targeting CD1d in CLL, because downregulation of CD1d as an evasion mechanism would be expected to have little or no effect on the continued survival and proliferation of the leukemic cells.

iNKT cells have been implicated in both promoting humoral immune responses to exogenous antigen and in suppressing B cell autoimmunity [10–12]. Because dnRAG1 and DTG mice show pronounced hypogammaglobulinemia associated with a defect in receptor editing, and because CD5^+^ B cells accumulating in these animals have a CD1d^hi^ immunophenotype, we reasoned that iNKT cells may be responsible for suppressing antibody production by B cells developing in these mouse strains. However, loss of iNKT cells did not significantly alter IgM or IgG levels in naïve dnRAG1 and DTG mice, suggesting that the suppression of antibody production in these animals is enforced by other mechanisms. Interestingly, however, serum IgG (but not IgM) levels were significantly reduced in naïve Eμ-TCL1 mice on a *Cd1d*^del/del^ background compared to those on a *Cd1d*^+/+^ background at 12 weeks of age, and this trend was also evident at the 36 week time point. Thus, in Eμ-TCL1 mice, iNKT cells appear to promote IgG production in the absence of exogenous antigenic stimulation, reminiscent to what has been reported in vitro in SLE patients and murine models of this disease [13, 14]. The observation that an iNKT cell-dependent reduction in IgG levels was not observed in naïve wild-type mice argues that different mechanisms regulate IgG production in naïve wild-type and Eμ-TCL1 mice.

To our knowledge, there are no previous reports describing B cell developmental defects in *Cd1d*^*−/−*^ mice. Consistent with this observation, no significant differences in the absolute numbers of cells in various developmental and mature B cell subsets were identified between WT *Cd1d*^*+/+*^ and WT *Cd1d*^*del/del*^ mice at 12 weeks of age. Interestingly, however, we noted a significant decrease in the absolute number of splenic transitional (B220^+^AA4.1^+^) B cells in all three transgenic strains of mice on a *Cd1d*^*del/del*^ background compared to their counterparts on a *Cd1d*^*+/+*^ background, particularly manifested at the transitional T1 stage. This trend was also noted in 36 week-old animals, but the differences were not statistically significant, possibly due to confounding disease progression in Eμ-TCL1 and DTG mice. Because dnRAG1, Eμ-TCL1, and DTG mice are all predisposed to accumulating CD5^+^ B cells over time [16, 18, 19], and CD5^+^ B cells in general have a predilection toward auto- and poly-reactivity [28], particularly against membrane-associated antigens which includes lipid-based molecules that can be presented on CD1d [28], these data raise the possibility that iNKT cells play a previously unrecognized role in mediating tolerance induction in developing B cells reactive toward membrane-associated lipid antigens. The reduced numbers of splenic transitional B cells in transgenic mice bred onto a *Cd1d*^*del/del*^ background suggests the normal role for the iNKT cells in this context is to provide pro-survival signals to transitional B cells reactive toward membrane-associated antigens, perhaps to facilitate receptor editing at the immature B cell stage of development to rescue B cell autoreactivity.

The increase in splenic T cell numbers, particularly CD8^+^ T cells, in 36 week-old *Cd1d*^*del/del*^ DTG mice compared to *Cd1d*^*+/+*^ DTG mice was unexpected, and raises the possibility that CD1d expression and/or iNKT cells regulate splenic T cell expansion in DTG mice during disease progression. There is some precedence for the role of iNKT cells in this process. Goubier et al*.* reported that iNKT cell deficiency enhanced skin inflammation in a model of contact hypersensitivity associated with increased recruitment of effector CD8^+^ T cells into antigen-exposed skin [29]. In addition, Ho et al*.* showed that activation of iNKT cells could suppress the proliferation of antigen-specific CD8^+^ T cells in vitro [30]. Finally, in a model of *Listeria monocytogenes* infection, Han et al*.* provided evidence that a hepatic population of NKT cells can suppress CD4^+^ and CD8^+^ T cell proliferation without interfering with their function in vitro and in vivo [31]. Taken together, these data suggest a model in which iNKT cells help restrain T cell proliferation in DTG mice as leukemia progresses. The observation that 36 week-old *Cd1d*^*del/del*^ and *Cd1d*^*+/+*^ DTG mice have similar absolute numbers of CD5^+^ B cells tends to reinforce this model by arguing against the possibility that the expanded populations of T cells in *Cd1d*^*del/del*^ DTG mice are contributing to greater clearance of leukemic CD5^+^ B cells in these animals. Interestingly, reminiscent of *Cd1d*^*del/del*^ DTG mice, human CLL patients often show a paradoxical elevation in the absolute number of T cells, due mainly to an increase of CD8^+^ T cells [32]. This observation has led to the suggestion that these T cells foster an environment to promote leukemic cell growth or survival. The data presented here raise the possibility that in human CLL, iNKT cells are responsible for restraining T cell proliferation to control CLL progression, which would be consistent with reports correlating loss of NKT-like cells with disease progression in human CLL [25, 26].

### Conclusions

Here we show that, similar to CLL-like B cells in Eμ-TCL1 mice, CD1d^hi^CD5^+^ B cells accumulating in dnRAG1 and DTG mice are IL10-competent. However, in all three murine models, loss of CD1d expression and iNKT cells had no effect on CD5^+^ B cell development, accumulation, or IL10 competence. These results imply that strategies to therapeutically target CD1d in CLL could be evaded by downregulating CD1d expression with little effect on continued leukemic cell survival. On the other hand, our findings associating the loss of CD1d expression and iNKT cells with a reduction in transitional B cell numbers in all three strains of transgenic mice, the suppression of spontaneous IgG production in Eμ-TCL1 mice, and the elevation of T cell numbers and an inversion of the CD4:CD8 T cell ratio in older DTG mice reveal new roles for iNKT cells in promoting progression of potentially polyreactive B cells through the transitional stage of B cell development, stimulating IgG production in Eμ-TCL1 mice, and restraining CD8^+^ T cell expansion in DTG mice. The latter observation may help explain reported associations in CLL between disease progression, loss of NKT-like cells, and a paradoxical increase in CD8^+^ T cells.

## Additional files

Additional file 1:
**Supporting Methods and Supporting References.** (PDF 98 kb) Additional file 2: Table S1. Bone marrow and splenic lymphocyte populations in 12 week-old WT, dnRAG1, Eμ-TCL1, and DTG mice on *Cd1d* ^+/+^ or *Cd1d* ^del/del^ backgrounds. (PDF 55 kb) Additional file 3: Figure S1.
*Cd1d* ^*del/del*^ mice at 12 and 36 weeks of age show loss of CD1d expression on B cells and an absence of iNKT cells. (PDF 214 kb) Additional file 4: Table S2. Bone marrow and splenic lymphocyte populations in 36 week-old WT, dnRAG1, Eμ-TCL1, and DTG mice on *Cd1d* ^+/+^ or *Cd1d* ^del/del^ backgrounds. (PDF 55 kb)

## Abbreviations

B-CLPD: B cell chronic lymphoproliferative disorder
CLL: Chronic lymphocytic leukemia
dnRAG1 mice: Transgenic mice expressing catalytically inactive RAG1
DTG mice: dnRAG1xEμ-TCL1 double transgenic mice
Eμ-TCL1 mice: Transgenic mice expressing human T cell leukemia-1
ELISA: Enzyme-linked immunosorbent assay
iNKT cell: Invariant natural killer T cell
SLE: Systemic lupus erythematosus

## References

[1] Porcelli SA, Modlin RL (1999). The CD1 system: antigen-presenting molecules for T cell recognition of lipids and glycolipids. Annu Rev Immunol.

[2] Brossay L, Jullien D, Cardell S, Sydora BC, Burdin N, Modlin RL (1997). Mouse CD1 is mainly expressed on hemopoietic-derived cells. J Immunol.

[3] Amano M, Baumgarth N, Dick MD, Brossay L, Kronenberg M, Herzenberg LA (1998). CD1 expression defines subsets of follicular and marginal zone B cells in the spleen: beta 2-microglobulin-dependent and independent forms. J Immunol.

[4] Yanaba K, Bouaziz J-D, Haas KM, Poe JC, Fujimoto M, Tedder TF (2008). A regulatory B cell subset with a unique CD1dhiCD5+ phenotype controls T cell-dependent inflammatory responses. Immunity.

[5] Kotsianidis I, Nakou E, Spanoudakis E, Bouchliou I, Moustakidis E, Miltiades P (2011). The Diagnostic value of CD1d expression in a large cohort of patients with B-cell chronic lymphoproliferative disorders. Am J Clin Pathol.

[6] Fais F, Morabito F, Stelitano C, Callea V, Zanardi S, Scudeletti M (2004). CD1d is expressed on B-chronic lymphocytic leukemia cells and mediates alpha-galactosylceramide presentation to natural killer T lymphocytes. Int J Cancer.

[7] Bojarska-Junak A, Hus I, Dmoszynska A, Rolinski J (2014). CD1d expression is higher in chronic lymphocytic leukemia patients with unfavorable prognosis. Leuk Res.

[8] DiLillo DJ, Weinberg JB, Yoshizaki A, Horikawa M, Bryant JM, Iwata Y (2013). Chronic lymphocytic leukemia and regulatory B cells share IL-10 competence and immunosuppressive function. Leukemia.

[9] Van Kaer L, Parekh VV, Wu L (2013). Invariant natural killer T cells as sensors and managers of inflammation. Trends Immunol.

[10] Lang ML (2009). How do natural killer T cells help B cells?. Expert Rev Vaccines.

[11] Yang J-Q, Wen X, Kim PJ, Singh RR (2011). Invariant NKT cells inhibit autoreactive B cells in a contact- and CD1d-dependent manner. J Immunol.

[12] Wermeling F, Lind SM, Jordo ED, Cardell SL, Karlsson MC (2010). Invariant NKT cells limit activation of autoreactive CD1d-positive B cells. J Exp Med.

[13] Shen L, Zhang H, Caimol M, Benike CJ, Chakravarty EF, Strober S (2015). Invariant natural killer T cells in lupus patients promote IgG and IgG autoantibody production. Eur J Immunol.

[14] Takahashi T, Strober S (2008). Natural killer T cells and innate immune B cells from lupus-prone NZB/W mice interact to generate IgM and IgG autoantibodies. Eur J Immunol.

[15] Mendiratta SK, Martin WD, Hong S, Boesteanu A, Joyce S, VanKaer L (1997). CD1d1 mutant mice are deficient in natural T cells that promptly produce IL-4. Immunity.

[16] Hassaballa AE, Palmer VL, Anderson DK, Kassmeier MD, Nganga VK, Parks KW (2011). Accumulation of B1-like B cells in transgenic mice over-expressing catalytically inactive RAG1 in the periphery. Immunology.

[17] Scarfo L, Dagklis A, Scielzo C, Fazi C, Ghia P (2010). CLL-like monoclonal B-cell lymphocytosis: are we all bound to have it?. Semin Cancer Biol.

[18] Nganga VK, Palmer VL, Naushad H, Kassmeier MD, Anderson DK, Perry GA (2013). Accelerated progression of chronic lymphocytic leukemia in E mu-TCL1 mice expressing catalytically inactive RAG1. Blood.

[19] Bichi R, Shinton SA, Martin ES, Koval A, Calin GA, Cesari R (2002). Human chronic lymphocytic leukemia modeled in mouse by targeted TCL1 expression. Proc Natl Acad Sci U S A.

[20] Bai L, Constantinides MG, Thomas SY, Reboulet R, Meng F, Koentgen F (2012). Distinct APCs explain the cytokine bias of alpha-galactosylceramide variants in vivo. J Immunol.

[21] Park SH, Roark JH, Bendelac A (1998). Tissue-specific recognition of mouse CD1 molecules. J Immunol.

[22] Hobeika E, Thiemann S, Storch B, Jumaa H, Nielsen PJ, Pelanda R (2006). Testing gene function early in the B cell lineage in mb1-cre mice. Proc Natl Acad Sci U S A.

[23] Matsushita T, Yanaba K, Bouaziz J-D, Fujimoto M, Tedder TF (2008). Regulatory B cells inhibit EAE initiation in mice while other B cells promote disease progression. J Clin Invest.

[24] DiLillo DJ, Matsushita T, Tedder TF (2010). B10 cells and regulatory B cells balance immune responses during inflammation, autoimmunity, and cancer. Ann N Y Acad Sci.

[25] Bojarska-Junak A, Hus I, Sieklucka M, Wasik-Szczepanek E, Mazurkiewicz T, Polak P (2010). Natural killer-like T CD3+/CD16 + CD56+ cells in chronic lymphocytic leukemia: Intracellular cytokine expression and relationship with clinical outcome. Oncol Rep.

[26] Jadidi-Niaragh F, Jeddi-Tehrani M, Ansaripour B, Razavi SM, Sharifian RA, Shokri F (2012). Reduced frequency of NKT-like cells in patients with progressive chronic lymphocytic leukemia. Med Oncol.

[27] Weinkove R, Brooks CR, Carter JM, Hermans IF, Ronchese F (2013). Functional invariant natural killer T-cell and CD1d axis in chronic lymphocytic leukemia: implications for immunotherapy. Haematologica.

[28] Hardy RR, Hayakawa K (1994). CD5 B cells, a fetal B cell lineage. Adv Immunol.

[29] Goubier A, Vocanson M, Macari C, Poyet G, Herbelin A, Nicolas JF (2013). Invariant NKT cells suppress CD8(+) T-Cell-mediated allergic contact dermatitis independently of regulatory CD4(+) T cells. J Investig Dermatol.

[30] Ho LP, Urban BC, Jones L, Ogg GS, McMichael AJ (2004). CD4(−)CD8 alpha alpha subset of CD1d-restricted NKT cells controls T cell expansion. J Immunol.

[31] Han YM, Jiang ZP, Chen ZB, Gu Y, Liu YF, Zhang X (2015). Pathogen-expanded CD11b(+) invariant NKT cells feedback inhibit T cell proliferation via membrane-bound TGF-beta 1. J Autoimmun.

[32] Mellstedt H, Choudhury A (2006). T and B cells in B-chronic lymphocytic leukaemia: Faust, Mephistopheles and the pact with the Devil. Cancer Immunol Immunother.

