# Supplementary material for: Cd1d regulates B cell development but not B cell accumulation and IL10 production in mice with pathologic CD5+ B cell expansion
Source: BMC Immunol. 2015 Nov 4;16:66. doi: 10.1186/s12865-015-0130-z (PMC4632344; doi:10.1186/s12865-015-0130-z)
Supplement: Additional file 1: — Supporting Methods and Supporting References. (PDF 98 kb) [file 12865_2015_130_MOESM1_ESM.pdf]

Cd1d regulates B cell development but not B cell accumulation and IL10 production in mice with pathologic CD5^{+} B cell expansion

Victoria L. Palmer, Vincent K. Nganga, Mary E. Rothermund, Greg A. Perry, and Patrick C. Swanson

SUPPORTING METHODS

Mice

Genotyping for the dnRAG1, Eμ-TCL1, and mb1-Cre transgenes was performed as described [1-3]. *Cd1d*^{fl} mice were genotyped by PCR to detect the 3' *loxP* site (CD1d Flox For, 5′-TTT GCA TTA ATG ACT GAA AGG TG-3′; CD1d Flox Rev, 5′- CTT GGG GTC CAT TCC AGA TA-3′). PCR amplified a 368-bp fragment in mice with *loxP* sites, compared to a 173-bp fragment detected in wild-type mice. PCR frequently failed to amplify the *loxP* site when offspring resulting from mating *Cd1d*$^{fl/fl}$ mice and mb1-Cre mice on dnRAG1 or Eμ-TCL1 backgrounds in various configurations were genotyped. Germline deletion of the conditional allele (called *Cd1d*^{del}) was confirmed by PCR using a forward primer specific for exon 1 (CD1d Exon 1 For, 5′-CCA TGG CTG TTG CTG TGG GC-3′) and the CD1d Flox Rev primer, which yielded an ~350 bp fragment. The *Cd1d*^{del} allele was transmitted to subsequent offspring, even if they lacked the mb1-Cre transgene, so these animals were used for subsequent matings to eventually obtain dnRAG1 *Cd1d*$^{del/del}$ and Eμ-TCL1 *Cd1d*$^{del/del}$ breeders (all lacking the mb1-Cre transgene) that we used to obtain cohorts of *Cd1d*$^{del/del}$ WT, dnRAG1, Eμ-TCL1, and DTG mice.

Flow cytometry

Single-cell suspensions prepared from spleen and bone marrow were depleted of red blood cells by hypotonic lysis and stained with fluorochrome-conjugated antibodies as previously described. The following antibodies were used: BD Biosciences (San Jose, CA) anti-B220-PE-CF594 (RA3-6B2), anti-CD19-APC-Cy7 (ID3), anti-CD4-APC-Cy7 (GK1.5), anti-CD23-Biotin (B3B4), anti-CD43-Biotin (S7), anti-CD212-Biotin (114), anti-Ly6C-PerCP-Cy5.5 (AL-21), and anti-NK-1.1-PE-Cy7 (PK136), and Biolegend (San Diego, CA) anti-CD1d-PerCP-Cy5.5 (1B1), anti-CD21-APC-Cy7 (7E9), and eBioscience (San Diego, CA) anti-CD3e-APC (145-2C11), anti-CD4-A700 (GK1.5), anti-CD5-PE (53-7.3), CD8-A700 (53-6.7), CD19-A700 (1D3), CD49b-PE-Cy7 (DX5), CD93-PE (AA4.1), CD93-PE-Cy7 (AA4.1), anti-IgM-APC (II/41), anti-IgM-PE-Cy5 (II/41), anti-IgD-FITC (11-26c), and Southern Biotech (Birmingham, AL) anti-CD24-Spectral Red (91). PE-conjugated CD1d tetramers, unloaded or loaded with PBS-57, were obtained from the NIH Tetramer Facility. Samples stained with biotinylated antibodies were detected using streptavidin-BUV737 (BD Biosciences San Jose, CA). Data collection was performed using a FACSAria flow cytometer (BD Biosciences). Data was analyzed using the FlowJo software (Tree Star, Inc. Ashland, OR).

SUPPORTING REFERENCES

1. Hassaballa, A.E., Palmer, V.L., Anderson, D.K., Kassmeier, M.D., Nganga, V.K., Parks, K.W., Volkmer, D.L., Perry, G.A., Swanson, P.C. (2011) Accumulation of B1-like B cells in transgenic mice over-expressing catalytically inactive RAG1 in the periphery. *Immunology* **134**, 469-86.
2. Bichi, R., Shinton, S.A., Martin, E.S., Koval, A., Calin, G.A., Cesari, R., Russo, G., Hardy, R.R., Croce, C.M. (2002) Human chronic lymphocytic leukemia modeled in mouse by targeted TCL1 expression. *Proc Natl Acad Sci U S A* **99**, 6955-60.
3. Hobeika, E., Thiemann, S., Storch, B., Jumaa, H., Nielsen, P.J., Pelanda, R., Reth, M. (2006) Testing gene function early in the B cell lineage in mb1-cre mice. *Proc Natl Acad Sci U S A* **103**, 13789-94.
