# Supplementary material for: Cd1d regulates B cell development but not B cell accumulation and IL10 production in mice with pathologic CD5+ B cell expansion
Source: BMC Immunol. 2015 Nov 4;16:66. doi: 10.1186/s12865-015-0130-z (PMC4632344; doi:10.1186/s12865-015-0130-z)
Supplement: Additional file 2: Table S1. — Bone marrow and splenic lymphocyte populations in 12 week-old WT, dnRAG1, Eμ-TCL1, and DTG mice on Cd1d +/+ or Cd1d del/del backgrounds. (PDF 55 kb) [file 12865_2015_130_MOESM2_ESM.pdf]

Table S1. Bone marrow and spleen populations in 12 wk WT, dnRAG1, Eμ-TCL1, and DTG mice on *Cd1d*$^{+/+}$ or *Cd1d*$^{del/del}$ backgrounds

Source and Subset	WT *Cd1d*$^{+/+}$ (n=4)	dnRAG1 *Cd1d*$^{+/+}$ (n=4)	Eμ-TCL1 *Cd1d*$^{+/+}$ (n=4)	DTG *Cd1d*$^{+/+}$ (n=4)	WT *Cd1d*$^{del/del}$ (n=4)	dnRAG1 *Cd1d*$^{del/del}$ (n=4)	Eμ-TCL1 *Cd1d*$^{del/del}$ (n=4)	DTG *Cd1d*$^{del/del}$ (n=4)
Bone Marrow								
Total Cellularity (x10^{5})	325±61	372±53	388±39	433±59	366±38	318±17	352±63	357±26
Total Lymphocytes (x10^{5})	131±20	143±18	156±17	190±25	141±17	122±10	141±25	152±11
B220^{+}CD43^{+}IgM^{-} [a]	2.7±0.2	2.8±0.3	3.3±0.8	3.0±0.6	2.4±0.6	1.9±0.4	2.2±0.5	2.2±0.5
B220^{+}CD43^{-} [a]	42±3	44±5	56±3	54±4	48±8	31±3	57±13	42±3
Pre-B (IgM^{-} IgD^{-}) [b]	23±2	24±3	30±0.5	34±1	26±4	19±2	29±7	28±3
Immature (IgM^{+} IgD^{-}) [b]	7.2±0.8	6.9±1.6	8.4±0.4	7.7±0.1	8.4±1.3	5.2±0.6	9.3±2.4	6.3±1.1
Mature (IgM^{+} IgD^{+}) [b]	11±1	7.2±0.7	16±2	8.4±0.8	12±3	5.3±0.4	17±4	5.6±0.4
Spleen								
Total Cellularity (x10^{5})	470±52	524±48	465±91	950±138	479±52	514±84	360±70	865±136
Total Lymphocytes (x10^{5})	306±33	341±35	320±71	644±98	317±32	334±54	237±47	623±107
CD19^{+} [c]	116±15	143±7	117±17	413±65	102±16	150±19	84±18	417±72
B220^{hi}AA4.1^{+} CD5^{-} [d]	21±2	16±1	22±3	14±2	16±2	9.5±1.9*	12±3*	6.9±0.8*
T1 (IgM^{hi}CD23^{-}) [e]	9.8±1.1	9.9±1.0	12±2	9.7±1.3	8.0±0.9	6.0±1.3	6.8±1.9*	4.6±0.6*
T2 (IgM^{hi}CD23^{+}) [e]	5.6±0.3	1.8±0.1	5.0±0.8	0.84±0.15	4.1±0.7*	1.0±0.2	2.5±0.8*	0.38±0.07
T3 (IgM^{dim}CD23^{+}) [e]	2.6±0.3	0.89±0.08	2.3±0.3	0.46±0.05	1.9±0.2*	0.47±0.06	0.89±0.21*	0.21±0.03
B220^{hi}AA4.1^{-}CD5^{-} [d]	79±12	47±3	79±13	31±3	71±11	36±5	59±12	27±3
MZ (CD21^{hi}CD23^{-}) [f]	3.1±0.7	7.1±0.7	4.7±1.4	1.6±0.4	3.9±0.7	5.1±1.1	3.2±0.9	1.1±0.3
FM (CD21^{int}CD23^{int}) [f]	68±10	29±2	65±10	18±1	60±10	21±3	45±9	15±2
CD5^{+} [d]	8.2±0.5	71±5	8.9±1.1	349±63	7.5±1.5	98±13	8.3±1.5	371±73
B220^{-}CD3^{+} [c]	111±8	120±14	110±26	119±18	120±9	102±21	86±23	111±13
CD4^{+} [g]	63±5	74±10	62±15	72±10	67±6	62±13	48±13	69±9
CD8^{+} [g]	39±3	36±5	39±10	36±8	44±3	32±7	30±9	30±4
Total CD1d-tet^{+} [g]	1.5±0.1	2.7±0.5	1.2±0.2	2.0±0.4	0.02*	0.02*	<0.01*	0.02*
NK1.1^{+} CD1d-tet^{+} [h]	0.90±0.11	1.4±0.3	0.74±0.10	1.2±0.2	<0.01*	<0.01*	<0.01*	<0.01*
NK1.1^{-} CD1d-tet^{+} [h]	0.62±0.11	1.2±0.2	0.46±0.12	0.84±0.14	<0.02*	<0.02*	<0.01*	<0.02*

[a] Total cells (x 10^{5}) calculated from Ly6C^{-}DX5^{-}CD4^{-} cells within a lymphocyte gate.
[b] Total cells (x 10^{5}) calculated from gated B220^{+}CD43^{-} cells.
[c] Total cells (x 10^{5}) calculated from gated lymphocytes.
[d] Total cells (x 10^{5}) calculated from gated CD19^{+} cells.
[e] Total cells (x 10^{5}) calculated from gated B220^{hi}AA4.1^{+}CD5^{-} cells.
[f] Total cells (x 10^{5}) calculated from gated B220^{hi}AA4.1^{-} CD5^{-} cells
[g] Total cells (x 10^{5}) calculated from gated B220^{-} CD3^{+} lymphocytes.
[h] Total cells (x 10^{5}) calculated from gated CD1d-tet^{+} lymphocytes.
* $p < 0.05$ compared to similar *Cd1d*$^{+/+}$ counterpart (e.g. dnRAG1 *Cd1d*$^{del/del}$ vs dnRAG1 *Cd1d*$^{+/+}$).
