# Supplementary material for: Cd1d regulates B cell development but not B cell accumulation and IL10 production in mice with pathologic CD5+ B cell expansion
Source: BMC Immunol. 2015 Nov 4;16:66. doi: 10.1186/s12865-015-0130-z (PMC4632344; doi:10.1186/s12865-015-0130-z)
Supplement: Additional file 3: Figure S1. — Cd1d del/del mice at 12 and 36 weeks of age show loss of CD1d expression on B cells and an absence of iNKT cells. (PDF 214 kb) [file 12865_2015_130_MOESM3_ESM.pdf]

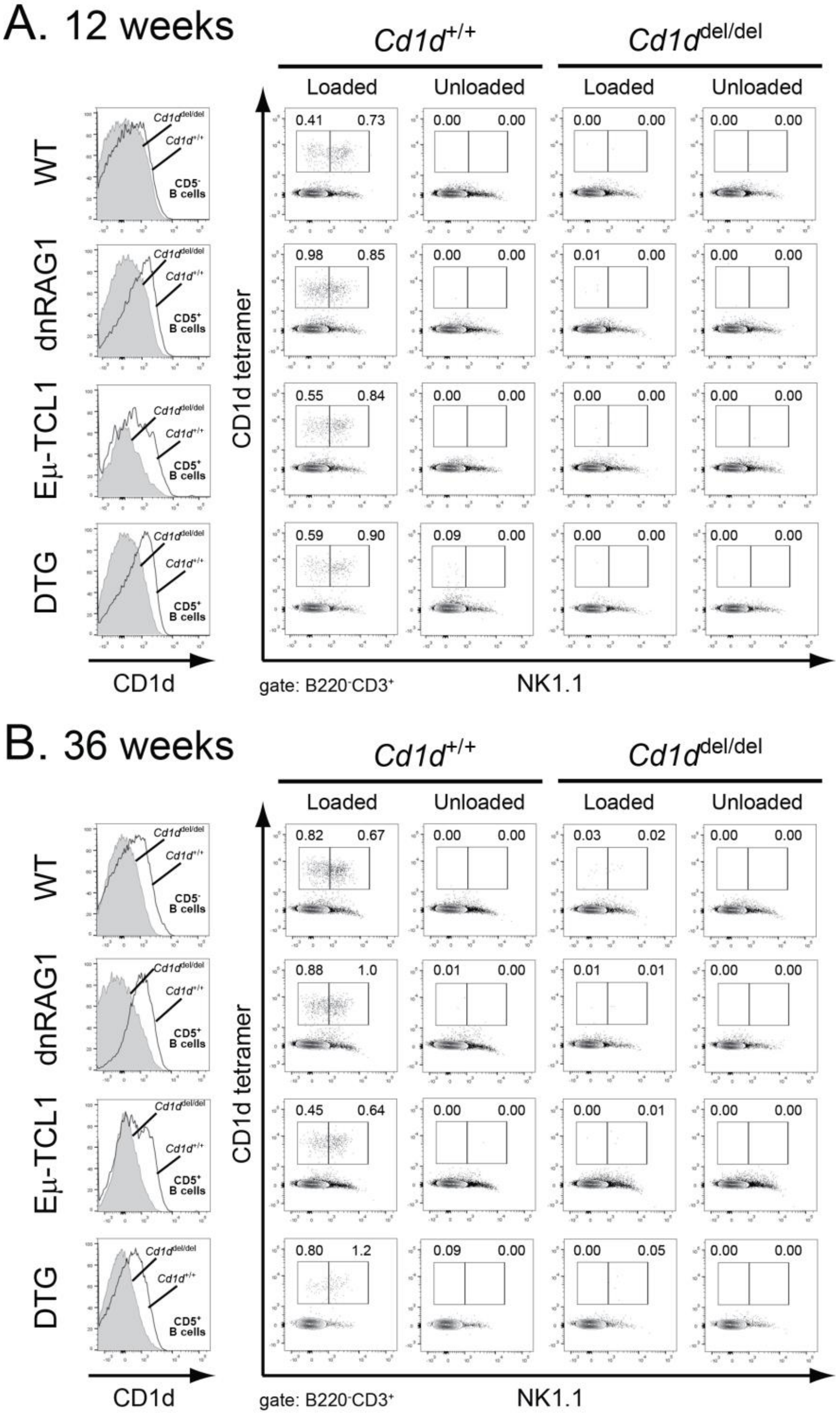

Figure S1. *Cd1d*$^{del/del}$ mice show loss of CD1d expression on B cells and an absence of CD1d-restricted NKT cells.

(A-B) WT, dnRAG1, Eμ-TCL1, and DTG mice on a *Cd1d*$^{+/+}$ or *Cd1d*$^{del/del}$ background were compared at 12 weeks (A) or 36 weeks (B) for the expression of CD1d expression on gated splenic CD19^{+} B cells that were either CD5^{-} (WT mice) or CD5^{+} (dnRAG1, Eμ-TCL1, and DTG mice) by flow cytometry (overlays, left panel), and for the presence of splenic CD1d-restricted NKT cells using flow cytometry by staining cells with CD1d tetramer (unloaded or loaded with PBS-57). Gated B220^{-}CD3^{+} cells were analyzed for NK1.1 expression on CD1d tetramer^{+} cells (right panels). The percentage of NK1.1^{-} and NK1.1^{+} cells in this population is shown for each genotype and is representative of n=4-6 animal/genotype. The mean absolute number of cells in each population is summarized in Table S1.
