# Supplementary material for: Cd1d regulates B cell development but not B cell accumulation and IL10 production in mice with pathologic CD5+ B cell expansion
Source: BMC Immunol. 2015 Nov 4;16:66. doi: 10.1186/s12865-015-0130-z (PMC4632344; doi:10.1186/s12865-015-0130-z)
Supplement: Additional file 4: Table S2. — Bone marrow and splenic lymphocyte populations in 36 week-old WT, dnRAG1, Eμ-TCL1, and DTG mice on Cd1d +/+ or Cd1d del/del backgrounds. (PDF 55 kb) [file 12865_2015_130_MOESM4_ESM.pdf]

Table S2. Bone marrow and spleen populations in 36 wk WT, dnRAG1, Eμ-TCL1, and DTG mice on *Cd1d*$^{+/+}$ or *Cd1d*$^{del/del}$ backgrounds

**Source and Subset**	**WT** ***Cd1d***$^{+/+}$ (n=5)	**dnRAG1** ***Cd1d***$^{+/+}$ (n=5)	**Eμ-TCL1** ***Cd1d***$^{+/+}$ (n=5)	**DTG** ***Cd1d***$^{+/+}$ (n=5)	**WT** ***Cd1d***$^{del/del}$ (n=5)	**dnRAG1** ***Cd1d***$^{del/del}$ (n=6)	**Eμ-TCL1** ***Cd1d***$^{del/del}$ (n=4)	**DTG** ***Cd1d***$^{del/del}$ (n=5)
Bone Marrow								
Total Cellularity (x10^5)	414±25	494±43	445±43	424±37	408±29	496±36	308±93*	477±44
Total Lymphocytes (x10^5)	181±20	233±27	221±22	223±25	174±14	226±18	149±45	214±19
B220$^+$CD43$^+$IgM$^-$ [a]	3.4±0.4	3.5±0.4	4.0±0.4	2.8±0.3	3.3±0.4	3.8±0.4	2.7±0.9	2.8±0.7
B220$^+$CD43$^-$ [a]	39±7	52±12	57±9	49±10	36±5	52±6	24±8*	23±4*
Pre-B (IgM$^-$ IgD$^-$) [b]	19±5	31±9	34±6	24±6	16±4	28±5	14±5*	12±3
Immature (IgM$^+$ IgD$^-$) [b]	5.5±1.5	8.7±2.4	9.7±2.4	11±5	4.0±0.9	8.1±1.4	3.3±1.2	3.5±0.9*
Mature (IgM$^+$ IgD$^+$) [b]	14±1	12±1	12±2	12±3	15±1	14±1	5.3±2.3*	6.4±2.2*
Spleen								
Total Cellularity (x10^5)	597±60	481±80	647±82	7173±3620	507±39	661±48	2324±1242	8971±3216
Total Lymphocytes (x10^5)	356±42	299±57	392±39	5298±3053	271±33	424±30	1318±730	6153±2427
CD19$^+$ [c]	127±15	140±24	155±22	4567±2963	93±20	214±25	800±575	4457±2004
B220^{hi}AA4.1$^+$ CD5$^-$ [d]	14±2	9.6±2.0	14±1	20±7	7.0±0.1	8.5±0.6	11±4	17±8
T1 (IgM^{hi}CD23$^-$) [e]	5.7±1.1	5.4±1.1	7.9±0.6	14±5	2.4±0.2	4.7±0.4	5.8±2.3	10±5
T2 (IgM^{hi}CD23$^+$) [e]	5.6±0.9	2.3±0.6	2.8±0.4	0.91±0.21	2.8±0.1*	1.9±0.2	1.3±0.5	1.4±0.9
T3 (IgM^{dim}CD23$^+$) [e]	1.6±0.2	0.9±0.2	1.1±0.2	0.31±0.07	1.2±0.2	0.79±0.07	0.55±0.13	0.53±0.44
B220^{hi}AA4.1$^-$CD5$^-$ [d]	107±13	100±17	117±19	1485±878	83±20	148±17	540±367	1177±419
MZ (CD21^{hi}CD23$^-$) [f]	6.2±0.6	5.1±1.2	2.5±1.5	6.8±5.9	5.1±0.8	9.8±2.2	1.1±0.4	1.2±0.7
FM (CD21^{int}CD23^{int}) [f]	91±10	75±10	91±16	576±389	68±17	104±13	308±209	273±152
CD5$^+$ [d]	10±1	75±16	86±19	4439±2953	7.1±1.0	133±23	730±558	4256±1978
B220$^-$CD3$^+$ [c]	135±18	90±22	126±19	156±28	105±14	120±9	182±41	430±125*
CD4$^+$ [g]	74±10	50±12	63±8	77±11	57±7	63±5	84±20	119±20*
CD8$^+$ [g]	48±7	31±8	49±9	51±12	37±7	46±4	66±10	239±115*
Total CD1d-tet$^+$ [g]	2.6±0.5	2.0±0.4	2.1±0.5	4.6±1.2	0.060±0.017*	0.051±0.019*	0.035±0.014*	0.11±0.04*
NK1.1$^+$ CD1d-tet$^+$ [h]	1.5±0.8	1.0±0.2	0.96±0.24	2.7±0.7	0.020±0.005*	0.018±0.004*	0.014±0.006*	0.035±0.016*
NK1.1$^-$ CD1d-tet$^+$ [h]	1.1±0.2	0.95±0.21	1.1±0.2	2.0±0.7	0.040±0.013*	0.033±0.015*	0.021±0.010*	0.076±0.029*

[a] Total cells (x 10^5) calculated from Ly6C$^-$DX5$^-$CD4$^-$ cells within a lymphocyte gate.
[b] Total cells (x 10^5) calculated from gated B220$^+$CD43$^-$ cells.
[c] Total cells (x 10^5) calculated from gated lymphocytes.
[d] Total cells (x 10^5) calculated from gated CD19$^+$ cells.
[e] Total cells (x 10^5) calculated from gated B220^{hi}AA4.1$^+$CD5$^-$ cells.
[f] Total cells (x 10^5) calculated from gated B220^{hi}AA4.1$^-$ CD5$^-$ cells
[g] Total cells (x 10^5) calculated from gated B220$^-$ CD3$^+$ lymphocytes.
[h] Total cells (x 10^5) calculated from gated CD1d-tet$^+$ lymphocytes.
* $p < 0.05$ compared to similar *Cd1d*$^{+/+}$ counterpart (e.g. dnRAG1 *Cd1d*$^{del/del}$ vs dnRAG1 *Cd1d*$^{+/+}$).
